# Low penetrance of retinoblastoma for p.V654L mutation of the RB1 gene

**DOI:** 10.1186/1471-2350-12-76

**Published:** 2011-05-26

**Authors:** Chia-Cheng Hung, Shin-Yu Lin, Chien-Nan Lee, Chih-Ping Chen, Shuan-Pei Lin, Mei-Chyn Chao, Shyh-Shin Chiou, Yi-Ning Su

**Affiliations:** 1 Graduate Institute of Clinical Genomics, National Taiwan University College of Medicine, Taipei, Taiwan; 2 Department of Medical Genetics, National Taiwan University Hospital, Taipei, Taiwan; 3 Graduate Institute of Clinical Medicine, National Taiwan University College of Medicine, Taipei, Taiwan; 4 Department of Obstetrics and Gynecology, National Taiwan University Hospital, Taipei, Taiwan; 5 Department of Obstetrics and Gynecology, Mackay Memorial Hospital, Taipei, Taiwan; 6 Division of Genetics and Metabolism, Department of Pediatrics, Mackay Memorial Hospital, Taipei, Taiwan; 7 Division of Genetics, Endocrinology and Metabolism, Department of Pediatrics, Kaohsiung Medical University Hospital, Kaohsiung, Taiwan; 8 Department of Medical Genetics, College of Medicine, Kaohsiung Medical University, Kaohsiung, Taiwan; 9 Department of Pediatrics, Kaohsiung Medical University Hospital, Kaohsiung, Taiwan

## Abstract

**Background:**

Retinoblastoma is caused by compound heterozygosity or homozygosity of retinoblastoma gene (*RB1*) mutations. In germline retinoblastoma, mutations in the *RB1 *gene predispose individuals to increased cancer risks during development. These mutations segregate as autosomal dominant traits with high penetrance (90%).

**Methods:**

We screened 30 family members from one family using high resolution melting assay and DNA direct sequencing for mutations in the *RB1 *gene. We evaluate the phenotype and penetrance of germline mutations of the *RB1 *gene in a large Taiwanese family.

**Results:**

The molecular analysis and clinical details of this family showed phenotypic variability associated with the p.V654L mutation in exon 19 of the *RB1 *gene in 11 family members. The phenotype varied from asymptomatic to presence of a unilateral tumor. Only four individuals (2 males and 2 females) developed unilateral retinoblastoma, which resulted in calculated low penetrance of 36% (4/11). The four individuals with retinoblastoma were diagnosed before the age of three years. None of their relatives exhibited variable severity or bilateral retinoblastoma.

**Conclusions:**

The diseased-eye ratio for this family was 0.36, which is lower than current estimates. This suggests that the *RB1 *p.V654L mutation is a typical mutation associated with low penetrance.

## Background

Retinoblastoma (OMIM +180200) is a rare eye cancer of the developing retina in early childhood, typically before the age of five years, with an incidence of 1 in 20,000 live births [1,2]. Mutations of the retinoblastoma (*RB1*) gene, which is the first tumor suppressor gene located on chromosome 13q14, regulates the cell-cycle G1/S check point, resulting in either malignant retinoblastoma or benign retinoma [3]. The *RB1 *gene comprises 27 exons across 180 kb and transcribes 4.8 kb of messenger RNA (mRNA) into a protein of 928 amino acids [4]. Germline mutations in *RB1 *gene predispose carriers to retinoblastoma tumors, resulting from loss of heterozygosity [5], which is described in Knudson’s two-hit hypothesis [6]. Due to its high penetrance (over 90%), hereditary retinoblastoma is transmitted as an autosomal dominant trait, which accounts for 40% of cases, presenting with bilateral retinoblastoma because of high expressivity. The remaining 60% of *RB1 *non-intermediary germline mutations often present as sporadic cases of unilateral retinoblastoma, without family history of retinoblastoma [7].

To date, a total of 2,508 *RB1 *DNA variants have been registered in the Leiden Open Variation Database (LOVD), including 1,790 substitutions, 502 deletions, 131 duplications, 53 insertions, 28 deletion/insertions 1 variants with 2 changes in 1 allele, and 3 complex variants. Although more than 90% of germline mutations in one *RB1 *allele in individuals may cause cancerous tumors of the retina, previous investigations show that some families have extremely low penetrance of retinoblastoma [8,9]. The low penetrance of *RB1 *mutations is due to reduced expressivity. The distinct mutations within the *RB1 *gene associated with low penetrance retinoblastoma include a point mutation at codon 661 of exon 20 [9,10], a 3-bp deletion in exon 16 that results in the deletion of Asn480 [9], a 4-kb deletion involving exons 24 and 25 [11], and a splicing mutation at the last base of exon 21 [12].

In this study, we performed clinical assessments and molecular analyses in a large Taiwanese family with retinoblastoma. Clinical assessment and molecular analyses included the high resolution melting assay followed by DNA direct sequencing in an apparently sporadic index case with unilateral retinoblastoma, which led to identification of the c.1960 G>T mutation in exon 19 of the *RB1 *gene in this family. The aim of the current study was to assess the phenotype and penetrance of the germline *RB1 *p.V654L mutation in all members of the family.

## Methods

### Subjects and DNA Extraction

All study DNA samples were obtained from the Department of Medical Genetics, National Taiwan University Hospital. Genetic counseling was provided to all participating family members and informed consent for participating in the study was obtained from each family member (or guardian) under the authority of the Institutional Ethics Committee of National Taiwan University Hospital. Members of this pedigree were counseled to identify both personal and family medical histories of retinoblastoma. Genomic DNA of 30 individuals was extracted from peripheral whole blood using the Chemagic DNA Blood Kit (Chemagen, Baesweiler, Germany) according to the manufacturer’s instructions.

Magnetic resonance imaging (MRI) or computerized tomography (CT) was performed to diagnosis only one tumor (unifocal) in only one eye or multiple tumors in one eye (multifocal), to examine only one eye affected (unilateral retinoblastoma) or both eyes affected (bilateral retinoblastoma), and to confirm the stage of retinoblastoma.

### Polymerase Chain Reaction (PCR)

The coding region of the *RB1 *gene was amplified based on GenBank NC_000013 as the reference sequence using specific forward (5'-atc ttt ccc agc ttg cat tt-3') and reverse primers (5'-cat gat ttg aac cca gtc agc-3'). PCR was performed in a total volume of 10 μL containing 50 ng of genomic DNA, 0.15 μM of each primer, 2X LightCycler 480 High Resolution Melting Master Mix (Roche Diagnostics, Mannheim, Germany) in 2.5 mM MgCl2 as provided by the manufacturer. PCR amplicons were amplified in a multiblock system thermocycler (ThermoHybaid, Ashford, UK) using a standard PCR program. The protocol consisted of an initial denaturation at 95°C for 10 min, followed by 35 cycles consisting of denaturation at 94°C for 30 s, annealing at 50°C for 30 s, and extension at 72°C for 45 s, and then a final extension step at 72°C for 10 min.

### High resolution Melting Assay

The melting step was appended to the amplification step (completely denatured at 95°C, cooled to 65°C at a thermal transition rate of 4.4°C/s) and then heated to 95°C at a thermal transition rate of 2.2°C/s with continuous fluorescence monitoring on the LightCycler 480 (Roche Diagnostics). The melting curve data were analyzed with the gene scanning module software, version 1.3 (Roche Diagnostics). The melting curves were then converted to melting peaks by plotting the negative derivative of the fluorescence with respect to temperature against temperature (–dF/dT vs temperature).

### DNA sequencing

PCR products were purified by solid-phase extraction and bi-directionally sequenced using the Taq DyeDeoxy Terminator Cycle Sequencing Kit (Applied Biosystems, Foster City, CA, USA). Sequencing reactions were separated on a PE Biosystems 373A/3100 sequencer.

## Results

**Figure 1:**
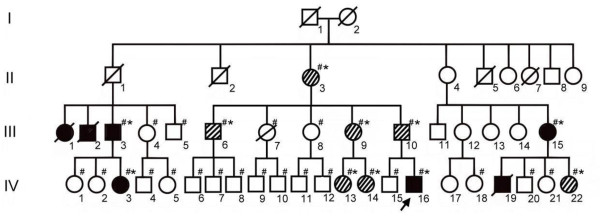
**The pedigree of a Taiwanese family with retinoblastoma**. The arrow shows the index case. The black symbols denote the individuals with unilateral retinoblastoma and the slanted lines denote the asymptomatic individuals with *RB1 *p.V654L mutations. The ‘#’ marks the family members who were tested, and ‘*’ marks individuals with the p.V654L mutation. The *RB1 *p.V654L mutation was identified in members II-3, III-3, III-6, III-9, III-10, III-15, IV-3, IV-13, IV-14, IV-16, and IV-22.

The index case, a 16-year old boy, was referred for a retinoblastoma evaluation at the age of 2 years (IV-16, Figure 1). The clinical diagnosis, made by MRI examination of the left eye (unilateral) showed the typical clinical features of retinoblastoma in the left eye. After comprehensive genetic counseling with the proband, the family history was positive for retinoblastoma. The pedigree of this large family is shown in Figure 1. Clinically, seven family members (III-1, III-2, III-3, III-15, IV-3, IV-16, and IV-19) developed unilateral retinoblastoma.

**Figure 2:**
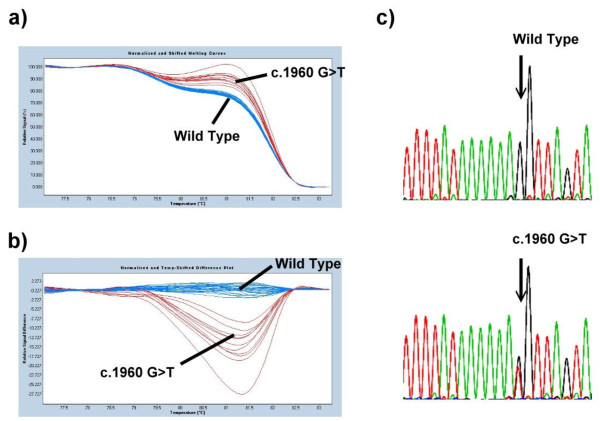
**The genetic analyses of RB1 gene**. (A) Exon 19 of the *RB1 *gene was analyzed in 30 family members and 20 unaffected individuals by high-resolution melting analysis. (B) After detection of the variant by high-resolution melting analysis, a derivative plot (-dF/dT vs temperature) of exon 19 of the *RB1 *gene was constructed. (C) Sequencing confirmed that the wild type cluster and the c.1960 G>T clusters were different.

Because the proband’s family history was positive for retinoblastoma, the detection rate of molecular genetic testing was at least 90%. Therefore, genotyping was performed on the proband by PCR amplification and subsequent high resolution melting assay. DNA analysis revealed a mutation in the *RB1 *gene, c.1960 G>T located on exon 19, a missense mutation, which resulted in a change in the residue from valine to leucine (Figure 2). This mutation was identified previously and is known to be pathogenic [12]. Subsequently, the 29 family members (the members denoted by ‘#’ in Figure 1) of the index case consented to participate in this study. Comprehensive mutations testing as well as retinoblastoma examinations were performed to identify both genotypes and phenotypes. Screening procedures in those at-risk family members showed that genotypes of 19 homozygous wild type (the members with # mark but without * mark) and an additional 10 heterozygous germ-line mutations (the members with # and * marks simultaneously in Figure 1) were identified and consented to participate in clinical examinations. Only four individuals, including the index case (two males and two females), developed unilateral retinoblastoma (the members in solid black), and seven asymptomatic subjects carry a germ-line mutation in one *RB1 *allele (the members with the slanted line). Furthermore, the ages of these seven mutation carriers without retinoblastoma are greater than 20 years of age. We presume the chance that these carriers will develop retinoblastoma later is low because retinoblastoma is rarely diagnosed after the age of 5 years and is typically diagnosed before the age of 3 years. Only a few cases of diagnosis after the age of 5 years are published.

The father of the index case (III-10), a 48-year old man, had the inherited *RB1 *p.V654L mutation, but without any suspicious clinical symptoms of retinoblastoma. Patients III-3, III-15, and VI-3, a 45-year old man, a 39-year old woman, and a 20-year old woman, are the uncle, aunt, and cousin of the index case, respectively, who had inherited the disease-causing mutation (p.V654L mutation). All of them had unilateral retinoblastoma in the left eye and were diagnosed with retinoblastoma before the age of 3 years. After surgical removal of the retinoblastomas, these affected individuals (including the index case) survived. In particular, none of them exhibited variable severity or bilateral retinoblastoma. In addition, they had no secondary tumors, especially osteosarcoma, which typically affects significant numbers of survivors of hereditary retinoblastoma.

The penetrance in this family is 4/11 (36%) among the available members and none of them has bilateral retinoblastoma, which is indicative of low expressivity for the germline *RB1 *c.1906 G>T mutation. The diseased-eye ratio for this family is 0.36, which is lower than typical of low penetrance retinoblastoma families [9].

## Discussion

This study describes a large Taiwanese family with retinoblastoma, with the germline *RB1 *p.V654L mutation. The family underwent comprehensive clinical examinations and genetic screenings, as well as genetic counseling. Clinical and genetic evaluations of the family members revealed that only four of the 11 individuals who carried the p.V654L mutation in one *RB1 *allele developed unilateral retinoblastoma, yielding a penetrance of only 36%. The ratio between symptomatic males and females is 1:1. The penetrance is lower than that of previous reports of individuals with *RB1 *null alleles. Although we did not analyze the expression of the p.V654L mutation at the RNA level, the reduced penetrance could be explained by a non-truncating mutation in which the resulting retinoblastoma protein is partially inactivated. The *RB1 *p.V654L mutation is located on the B pocket domain of the pRB protein, which forms a functional repressor motif with the A pocket domain, important in the retina. Many missense mutations are located in the domains A and B affecting its structural folding and stability. Because valine and leucine are both hydrophobic, non-polar amino acids, the missense is not likely to have a significant effect. The c.1960G > T mutation changes the final base of exon 19, and reduces the splicing score from 89 to 76.4 [13]. According to the previous study [14], the p.V654L mutation is in fact a splice mutation. The low-penetrance of c.1960G>C could result from the splicing mutation affecting only the last nucleotide of exon 19 [15]. The splicing machinery could alternate between the defective missense splicing (p.V654L mutation) and inactivation and skipping of exon 21 in the pocket box domain of *RB1*. This hypothesis could be why the penetrance of this mutation is so low in this particular family.

Speculative explanations for the low penetrance of retinoblastoma include the possibility that the incidence of somatic mutation, recombination, and non-disjunction, encourage the retention of the remaining normal allele in all retinal cells in carriers of a germ-line mutation [16]. Another possibility is that additional genetic events beyond the homozygous inactivation of the retinoblastoma gene are required to produce a retinoblastoma in some individuals, described as the three-hit theory [11]. Alternatively, in some families, there may be either a second retinoblastoma focus operating by a different mechanism to predispose weakly to this cancer or perhaps one or more modulator genes that reduce the risk in such carriers (host resistance factors) [17,18]. The weak-allele hypothesis [19] is that the inherited low penetrance retinoblastoma allele is partially, but not completely, impaired [20]. Loss of heterozygosity would result in two copies of the weak allele, which would still be sufficiently active to prevent tumorigenesis. Tumors would arise only when the second mutation is a null, resulting in a completely inactivated allele. Mutation affecting regulatory sequences in the *RB1 *promoter also reduce the expression of normal Rb protein below a certain threshold level necessary for tumor suppression function [21].

## Conclusions

In summary, we describe a large and extensively screened family with a germline *RB1 *p.V654L mutation. Though the underlying molecular mechanisms in cases of familial retinoblastoma with low penetrance and variable expressivity are not well understood, mutation identification permits accurate genetic counseling, without which the developing retinoblastoma tumors might be missed in children of families with low penetrance, screened clinically on the basis of conventional risk estimates.

## Competing interests

The authors declare that they have no competing interests.

## Authors' contributions

CCH and SYL performed the molecular genetics studies and drafted the manuscript. CNL, CPC, and SPL participated in the molecular genetics studies. MCC and SSC performed the clinical characterization of the patients. YNS conceived the study, participated in its design and coordination, and helped draft the manuscript. All authors read and approved the final manuscript.

## Pre-publication history

The pre-publication history for this paper can be accessed here:

http://www.biomedcentral.com/1471-2350/12/76/prepub
